# A School-Based Cross-Sectional Survey of Adverse Events following Co-Administration of Albendazole and Praziquantel for Preventive Chemotherapy against Urogenital Schistosomiasis and Soil-Transmitted Helminthiasis in Kwale County, Kenya

**DOI:** 10.1371/journal.pone.0088315

**Published:** 2014-02-10

**Authors:** Sammy M. Njenga, Paul M. Ng’ang’a, Mariam T. Mwanje, Fatuma S. Bendera, Moses J. Bockarie

**Affiliations:** 1 Eastern and Southern Africa Centre of International Parasite Control (ESACIPAC), Kenya Medical Research Institute (KEMRI), Mbagathi Road, Nairobi, Kenya; 2 Division of Vector-Borne Diseases & Neglected Tropical Diseases (DVBD-NTD), Ministry of Health, Nairobi, Kenya; 3 Environmental Health Department, Kwale County, Kenya; 4 Centre for Neglected Tropical Diseases, Liverpool School of Tropical Medicine, Liverpool, United Kingdom; Tulane University School of Public Health and Tropical Medicine, United States of America

## Abstract

**Background:**

Soil-transmitted helminths and schistosomiasis are mostly prevalent in developing countries due to poor sanitation and lack of adequate clean water. School-age children tend to be the target of chemotherapy-based control programmes because they carry the heaviest worm and egg burdens. The present study examines adverse events (AEs) experienced following co-administration of albendazole and praziquantel to school-age children in a rural area in Kwale County, Kenya.

**Methods:**

Children were treated with single doses of albendazole and praziquantel tablets and then interviewed using a questionnaire for post treatment AEs.

**Results:**

Overall, 752 children, 47.6% boys, participated in the study. Their median (interquartile range) age was 12.0 (10.0–14.0) years. A total of 190 (25.3%) children reportedly experienced at least one AE. In total, 239 cases of AEs were reported with the most frequent being abdominal pains (46.3%), dizziness (33.2%) and nausea (21.1%). Majority of the reported AEs (80.8%) resolved themselves while 12.1% and 6.3% were countered by, respectively, self-medication and visiting a nearby health facility. More girls (60.5%) than boys (39.5%) reported AEs (*P* = 0.027).

**Conclusions:**

The AEs were mild and transient, and were no worse than those expected following monotherapy. The current study adds to the evidence base that dual administration of albendazole and praziquantel in school-based mass drug administration is safe with only mild adverse events noted.

## Introduction

Soil-transmitted helminths (STH) and schistosomiasis are mostly prevalent in developing countries where poor sanitation and lack of adequate clean water are commonplace. STH infections are among the most prevalent infections in the world with around 1.4 billion individuals infected by *Ascaris lumbricoides*, 1.0 billion by *Trichuris trichiura*, and 1.3 billion by hookworms [1]. About 200 million people are estimated to be infected with schistosomiasis with 170 million living in sub-Saharan Africa while the remaining 30 million live in North Africa, Asia and South America [2]. The coastal areas of Kenya are highly endemic for urogenital schistosomiasis and STH [3], [4]. Children have the highest prevalence and intensity of STH and schistosomiasis infections, but the consequences of chronic infection, such as growth stunting, anaemia, hepatic/urinary fibrosis, and impaired cognitive development, continue to have an effect throughout adulthood [5].

In 2001, during the 54^th^ WHA, the WHO urged its member states to ensure provision for the regular antihelminthic treatment of all school-age children living in areas of schistosomiasis and STH endemicity [2]. Presently, schistosomiasis and STH control strategies focus on mass administration of praziquantel and albendazole, with special emphasis on treating school age children [6]. An integrated approach, against urogenital schistosomiasis and STH using praziquantel and albendazole, respectively, has been shown to be effective at reducing prevalence and intensity of infections in several countries in sub-Saharan Africa in various programmes [7], [8]. These orally administered drugs are affordable, efficacious and safe for the large-scale treatment of human populations [9], [10]. Further, they are given as single dose tablets and are therefore expected to ensure greater compliance [11]. School-aged children tend to be the target of chemotherapy-based control programmes because they carry the heaviest worm and egg burdens. This is due, in part, to their hygiene and behavioural practices, determining higher exposures and rates of acquisition of infection [12].Treatment of children is also likely to be more successful in averting the development of subsequent, more serious disease sequelae because earlier stages of infection-induced pathology may be reversible if treated promptly [13].

In 2009, a research project to evaluate a pilot schistosomiasis/STH control programme and its integration with the national LF elimination programme was established in Kwale District, Kwale County, Kenya. Kwale has previously been shown to be highly endemic for urogenital schistosomiasis and STH [14]–[16]. The aim of the present study was to determine the safety of co-administration of albendazole and praziquantel to school-age children for control of STH and urogenital schistosomiasis.

## Methods

### Ethical Statement

Permission to conduct the current study, including review and approval by the Scientific Steering and Ethical Review Committees, was obtained from Kenya Medical Research Institute (KEMRI). The district education office, district health office, local leaders, teachers and parents were informed about the study in the area. Written informed consent was obtained, from a guardian, caretaker or a parent, for each of the participants in the study. Besides, oral informed consent was obtained from the study participants. No respondent’s identifiers were included in the dataset used for these analyses. The dataset is available, for free, upon making a written request to the corresponding author.

### Study Site

The study was conducted in Mwaluphamba Location, Matuga District, Kwale County, Kenya. The district covers an area of 1,043 km^2^and has an estimated population of 151,978 residents [17]. The study area was previously described in a related publication by Njenga et al [14]. Mwaluphamba Location is situated in a rural setting of Matuga District bordering a drier Kinango District in the West. The district experiences a bimodal rainfall pattern with long rains occurring between mid-March and June while the short rains occur between October and December. The majority of the residents of the study area practice subsistence farming.

### Study Design and Population

The population involved in the present cross-sectional study included 752 school-age children from five sentinel schools, namely; Maponda (161), Miatsani (140), Mirihini (155), Kajiweni (167) and Mlafyeni (129).

### Treatment

In the month of July, 2010, children from the five schools received the first round of combination therapy. They were treated with a single dose of praziquantel (Prazitel®, Cosmos Ltd) using the dose pole method to determine the number of tablets to be administered to each child [9]. Additionally, the children were treated for soil-transmitted helminths using a single dose of albendazole 400 mg tablets (Unibazole®, Universal Pharmacy (K) Ltd). The programme, through the teachers, requested parents/guardians to provide a morning meal to the children on the day the MDA took place. Further, schoolteachers, who participated in the distribution of study drugs, were given a one day training facilitated by district health and education officers who were assisted by a national team from the Ministries of Health and Education and the research team from KEMRI.

### Data Collection and Statistical Analysis

One week after the administration of the medications, the children were interviewed for AEs using an interviewer-administered structured questionnaire. Details of the AEs experienced 48 hours post treatments, if any, were recorded. The reported AEs were classified as defined in the WHO guidelines whereby mild AEs were defined as undesirable experiences associated with use of the antihelmithics but not affecting daily activities (e.g. playing) whereas moderate AEs were defined as those affecting performance of daily activities. Severe AEs were defined as those requiring total rest and/or medication while serious AEs were defined as those that were life-threatening requiring admission to hospital [18], [19].

Data were entered into a computer using Microsoft Excel and then exported to IBM SPSS Statistics 19.0 for analyses. Appropriate descriptive statistics were computed. Chi-square (χ^2^) test was used to compare proportions. Statistical significance was set at *p*< 0.05.

## Results

A total of 752 school-aged children, 47.6% of whom were boys, were interviewed for AEs experienced following administration of albendazole and praziquantel tablets. Their median age was 12.0 years (interquartile range: 10.0 to 14.0 years) with a majority of them (70.7%) being in the age category of 10–14 years as shown in Table 1.

**Table 1:** Characteristics of the respondents.

Variable	Number (n = 752)	%
**Gender**		
Male	358	47.7
Female	393	52.3
No response	1	0.1
**Age category (years)**		
< 10	76	10.1
10 – 14	532	70.7
>14	144	19.1

**Figure 1:**
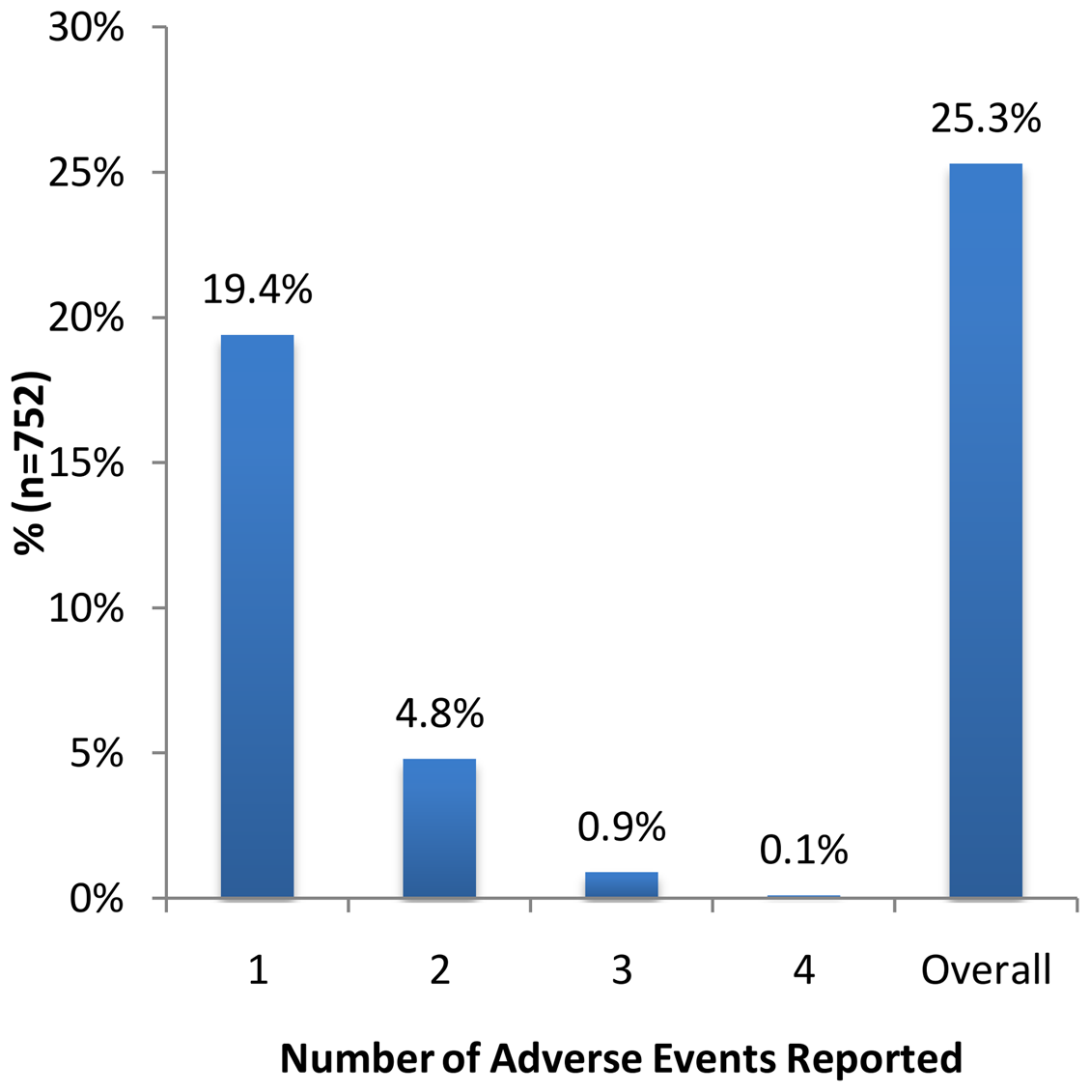
Distribution of the number of adverse events experienced.

After taking the medications, 190 (25.3%) children reported experiencing at least one AE as shown in Figure 1. The number of respondents who experienced one and two AEs were 146 (19.4%) and 36 (4.8%), respectively. Only eight respondents (1.0%) experienced more than two AEs.

Table 2 presents the description and frequencies of AEs reported by the school children interviewed. Overall, 239 cases of AEs were reported with abdominal pain being the most commonly reported AE. The other commonly reported AEs included dizziness, nausea, head ache and vomiting. Of the 239 AEs reported, majority (84.0%) lasted a day or less.

**Table 2:** Description and frequency of reported adverse events.

Description	Number (n = 239)	%
**Nature of adverse event**		
Abdominal pain	89	37.2
Dizziness	44	18.4
Nausea	40	16.7
Headache	33	13.8
Vomiting	10	4.2
Drowsiness	13	5.4
Urination*	5	2.1
Itching	5	2.1
**Duration of adverse events**		
1 day or less	199	83.3
More than a day	38	15.9
No response	2	0.8

*Polyuria (3) and dysuria (2).

The majority of the reported AEs were mild (166, 69.5%) while moderate and severe AEs constituted 23.8% (57) and 6.3% (15) of the total AEs reported respectively (Table 3). All the respondents who reported having experienced one or more AEs opined that the AEs were related to the study medications except for one respondent whose response was unavailable. Majority of the reported AEs (193, 80.8%) did not involve any intervention measures to mitigate those undesired events. Only 6.3% cases were reported to have involved seeking medical attention in a nearby hospital while 12.1% were countered by self-medication involving taking painkillers and anti-allergy drugs available in kiosks as Panadol® (paracetamol) and Piriton® (chlorpheniramine), respectively.

**Table 3:** Characteristics of the children who experienced adverse reactions.

Variable	No. (n = 190)	%	*p*-value
**Age (years)**			
<10	17	8.9	0.117
10 – 14	127	66.8	
>14	46	24.2	
**Sex**			
Male	75	39.5	0.027
Female	115	60.5	

Gender was significantly associated with AEs with more girls than boys reporting to have experienced AEs to the study drugs (60.5% versus 39.5%, *P* = 0.027). Investigations into the age categories showed that more children in the 10 to 14 years age category (66.8%) had had adverse reactions as compared to those in other age categories though this was not statistically significant (Table 3).

**Figure 2:**
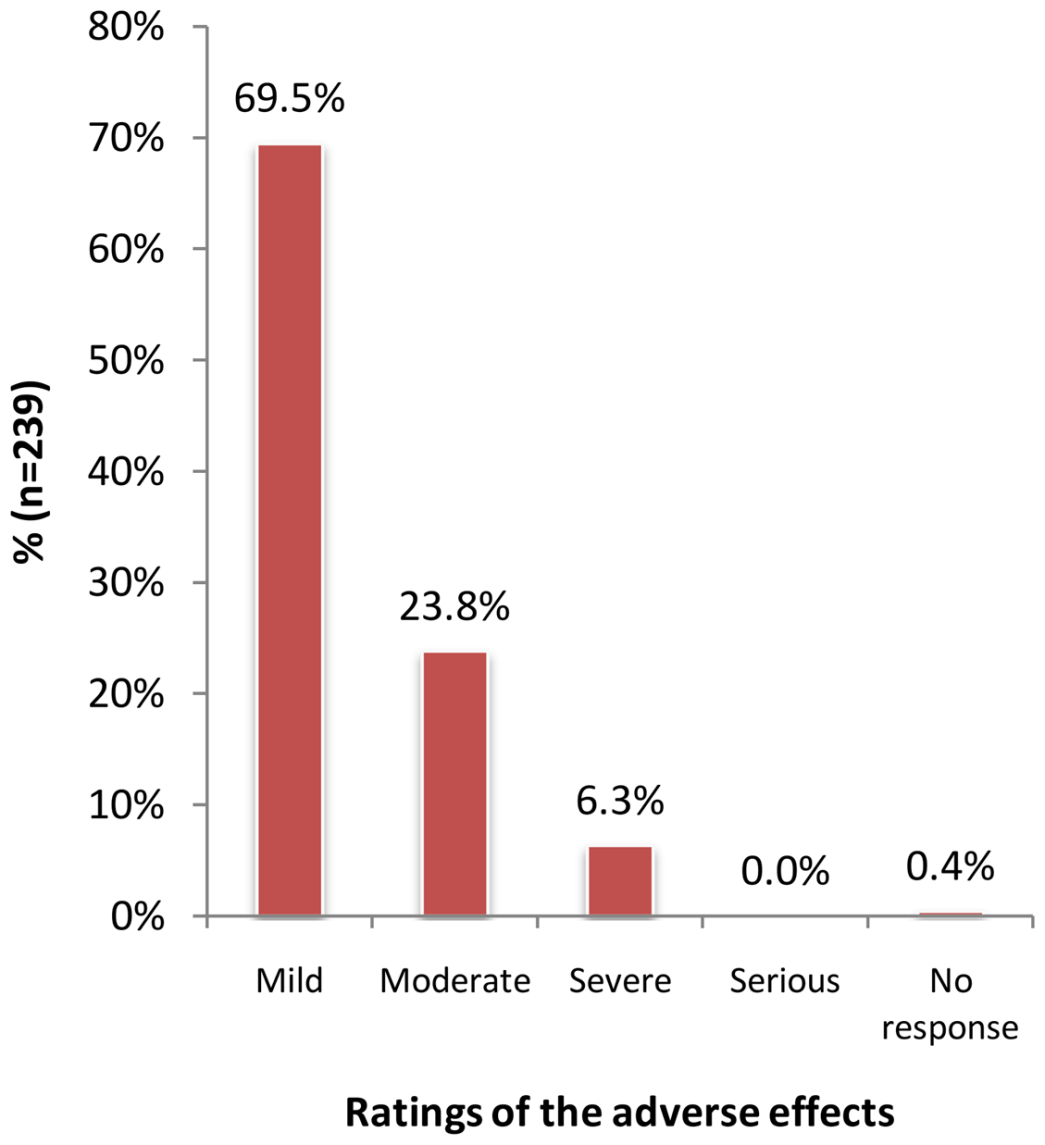
Severity of the adverse events.

Overall, no AE was rated by the respondents as serious while only a few AEs were rated as severe (23, 6.5%) as shown in Figure 2. The 23 cases of AEs were reportedly taken to hospital for treatment though no admissions were done. These severe AEs were constituted by vomiting (21.1%), stomachaches (11.9%) and headaches (8.9%).

Table 4 presents the relationship between gender and the type of AEs. Generally, more AEs were reported by girls than boys except for drowsiness. Nevertheless, this trend was not significant, statistically, but for dizziness (30.6% for boys versus 69.4% for girls, χ2 = 6.114, df =  1, p = 0.013).

**Table 4:** Association between adverse events and gender.

Adverse Event	Total(n = 752)	Male (n = 358)	Female (n = 393)	χ^2^	df	*P*-value
**Stomachache**						
Yes	88	41(46.6%)	47(53.4%)	0.047	1	0.829
No	663	317(47.8%)	346(52.2%)			
**Headache**						
Yes	33	13(39.4%)	20(60.6%)	0.948	1	0.330
No	718	345(48.1%)	373(51.9%)			
**Dizziness**						
Yes	49	15(30.6%)	34(69.4%)	6.114	1	0.013
No	702	343(48.9%)	359(51.1%)			
**Vomiting**						
Yes	10	3(30.0%)	7(70.0%)	1.268	1	0.260
No	741	355(47.9%)	386(52.1%)			
**Drowsiness**						
Yes	13	9(69.2%)	4(30.8%)	2.465	1	0.116
No	738	349(47.3%)	389(52.7%)			

## Discussion

A substantial proportion of children (25%) reported having experienced one or more AEs following co-administration of albendazole and praziquantel tablets. However, most of these AEs were mild and resolved within a day or two. The AEs reported in the current study included gastrointestinal disturbances such as abdominal pain, nausea and vomiting. Headaches, drowsiness and dizziness were also reported as AEs. Adoubryn et al [20] conducted a similar study in Biankouma, Côte d’Ivoire and found the frequency of AEs to be 40.8%, which is higher than in the current study. In another study, N’Goran et al found 33.3% of school children treated with praziquantel alone experienced one side effect AE or more which is also much higher compared to the present study [21]. A study on the side effects of praziquantel in a community of western Côte d’Ivoire, however, found a lower prevalence of side effects compared to the current study with only 12.5% of the individuals reporting one or more side effects 24 hours post-treatment [22]. The results of the current study are discordant with those from a study done in Southern Ethiopia where 83% of children treated with praziquantel alone complained that they experienced two or more treatment-related symptoms suggestive of side effects of the drug [23]. The reasons for the variations in proportions of AEs experienced after antihelminthic treatment could probably be due to dissimilar intensities of parasites as well as differences in socio-economic status, types of foods and environmental conditions associated with the diverse populations. Nevertheless, all the studies were in agreement with the current study by rating the side effects as mild.

By and large, the proportion of children who reportedly experienced AEs is comparable to what has been observed in other studies involving praziquantel whereby a higher proportion of respondents are reported to experience AEs as compared to albendazole. Indeed, a review by Horton (2000), reported that incidences of side effects associated with albendazole at the doses used for the treatment of intestinal helminths are very low, mild and self-limiting with only gastrointestinal side effects occurring with an overall frequency of just greater than 1% [24]. Side effects due to praziquantel, however, have been reported to occur in 30–60% of the patients, but, just like for albendazole, they are also mild and transient and disappear within 24 hours [21], [25], [26]. The most prevalent AE in the present study was abdominal pain (37.2%), followed by dizziness (18.4%) and nausea (16.7%). Other researchers have also found abdominal pain to be the most common AE reported after administration of praziquantel and/or albendazole. Studies conducted among school children in different parts of Kenya where intestinal schistosomiasis is endemic also found abdominal pain to be the most frequently reported AE following treatment [26], [27]. Further, a double-blind placebo-controlled study involving 11500 children from China, Kenya and Philippines concluded that AEs due to co-administrations of both albendazole and praziquantel are no worse than those observed with praziquantel alone and the main side effects are abdominal pain and headache [28].

The current study reported no serious life-threatening AEs. In fact, only 15(6.3%) respondents reported severe AEs while the rest were either were mild (69.5%) or moderate AEs (23.8%). The frequencies of side effects and also severity have been loosely linked to the proportion of the dying parasites and the subsequent release of their products [27], [29]. The abdominal pain may be related to a direct pharmacologic effect on the parasite, and possibly to a massive shift or death of worms in the mesenteric veins, which may lead to congestion and ultimately acute intestinal colic [29].

Significantly more girls than boys experienced AEs in the current study. A study by Raso et al using praziquantel alone reported abdominal pain to occur more frequently in women than men [17]. The reasons for differences in occurrence of AEs between males and females is unclear but could be due to physiological differences.

The current study’s limitations include the unavailability parasitological data which would have enriched the discussion since adverse effects are usually related to infection intensity. Moreover, having such data would allow comparisons with other published studies in the field. The delay before administering the interview seems too long, particularly given the age of the children and this may have lead to recall bias.

## Conclusions

Generally, a range of AEs were associated with the current drugs used in the treatment of schistosomiasis and STH, i.e., praziquantel and albendazole. This study, however, confirms that the safety of the two drugs even when co-administered. Approximately 6% of the children interviewed reported experiencing severe AEs related to the medications. Therefore, it is imperative that preventive chemotherapy intervention programmes put in place surveillance measures in order to ensure timely detection, management and reporting of potential life-threatening AEs. The current study adds to the evidence base that dual administration of albendazole and praziquantel in school-based MDA is safe with only mild adverse events (AEs) noted.
